# Effects of sound segregation cues on multi-sound intensity discrimination

**DOI:** 10.1121/10.0022559

**Published:** 2023-12-01

**Authors:** Kenta Watanabe, Ramesh Srinivasan, Virginia M. Richards

**Affiliations:** 1 Department of Cognitive Sciences, University of California, Irvine, 3151 Social Science Plaza, Irvine, California 92697, USA; 2 Department of Biomedical Engineering, University of California, Irvine, 3120 Natural Sciences II, Irvine, California 92697, USA kentaw1@uci.edu, srinivar@uci.edu, v.m.richards@uci.edu

## Abstract

The effects of sound segregation cues on the sensitivity to intensity increments were explored. Listeners indicated whether the second and fourth sounds (harmonic complexes) within a five-sound sequence were increased in intensity. The target sound had a fundamental frequency of 250 Hz. In different conditions, nontarget sounds had different fundamental frequencies, different spectral shapes, and unique frequency regions relative to the target. For targets more intense than nontargets, nontarget characteristics did not affect thresholds. For targets less intense than the nontargets, thresholds improved when the targets and nontargets had unique frequency regions.

## 1. Introduction

For a temporal series of short sounds, the more intense sounds may be overweighted when integrating information across the series of sounds. This general effect has been observed for frequency sample discrimination tasks (e.g., Berg, 1990) and multitone intensity discrimination tasks, where the intensity changes are applied across sequential sounds (e.g.,Oberfeld *et al.*, 2013; Ponsot *et al.*, 2013). Here, this general finding will be referred to as level dominance (Berg, 1990).

Whereas peripheral masking is expected to contribute to level dominance (e.g., forward masking; Zeng *et al.*, 1991), there is evidence that higher-level, central/cognitive processes also contribute. For example, in a multitone intensity discrimination task, Oberfeld *et al.* (2013) found that for some listeners, level dominance is associated with centrally generated noise. Additionally, sequential grouping of the maskers and targets into separate auditory objects (Oberfeld and Stahn, 2012) and perceived lateralization (Oberfeld *et al.*, 2012) led to a release from masking. For frequency sample discrimination tasks, Turner and Berg (2007) showed that level dominance is observed with inter-sound intervals well beyond 300 ms, which is unlikely to be attributable to forward or backward masking.

Lutfi and Jesteadt (2006) examined level dominance using a multitone intensity discrimination task. A series of sounds were presented such that their intensities varied high-low-high-low-high. The intensity increment applied to the lower-level tones was larger than that for the higher-level tones. Nonetheless, listeners overweight the information in the more intense sounds. However, when the higher-level tone pips were replaced by broadband noise, listeners more efficiently used the information from the lower-level tones, although the pattern of their weighting remained suboptimal. The authors suggest that this release from level dominance reflects the difference in the quality of the more versus less intense sounds.

The current experiments are somewhat parallel to those of Lutfi and Jesteadt (2006). A series of sounds with alternating levels were used, and the increment in level to be detected was applied to the second and fourth of five sounds. The goal is to evaluate the role of changes in sound “quality” on intensity discrimination. The makeup of the nontarget sounds was altered to systematically influence the difference in quality of the target and nontarget sounds. As a proxy for changes in quality, the makeup of the nontarget sounds varied from the target sounds along the continuum of changes that are expected to promote stream segregation or not. Notably, the target and nontarget sounds within the sequence were perceptibly different. However, they were heard as a sequence, not as two streams due to the timing between sounds and the small number of sounds (five) per trial.

In the first experiment, the composition of the higher- and lower-level sounds were varied using manipulations tested by Oh *et al.* (2022), who systematically evaluated the impact of changes in fundamental frequency and spectral slope on stream segregation (see also Bregman *et al.*, 1990; Vliegen and Oxenham, 1999; Moore and Gockel, 2002). The results indicate that neither differences in fundamental frequency nor spectral slope led to changes in intensity discrimination thresholds. In the second experiment, the spectral extent of the target and nontarget sounds differed, providing strong peripheral cues for differences between the two sounds. The results indicate consistent releases from masking for these conditions.

## 2. Methods

### 2.1 Participants

A total of 12 subjects participated in the experiments, half of whom participated in more than 1 experiment. Six participated in experiment 1 (aged 18–30 years old, four males), seven participated in experiment 2A (aged 18–29 years old, one male), and six participated in experiment 2B (aged 18–30 years old, three males). Only K.W. participated in all three experiments.^1^ The subjects were paid an hourly wage, except for K.W. All subjects had absolute thresholds of 20 dB hearing level (HL) or better for audiometric frequencies between 250 and 8000 Hz.

### 2.2 Stimuli and design

The stimuli were digitally generated using a sampling frequency of 44 100 Hz on a personal computer (PC), which also controlled the experimental procedure and data collection through custom-written software (The MathWorks, Inc., Natick, MA). The stimuli were presented diotically via a 24-bit soundcard (Envy 24 PCI audio controller, VIA Technologies, Inc., Taipei, Taiwan), a programmable attenuator and headphone buffer (PA4 and HB6, Tucker-Davis Technologies, Inc., Alachua, FL), and Sennheiser HD 600 headsets (Wennebostel, Germany). Each stimulus presentation was followed by visual feedback as to the correctness of the subject's response. The experiment was conducted in a double-walled, sound-attenuated booth.

**Fig. 1.**
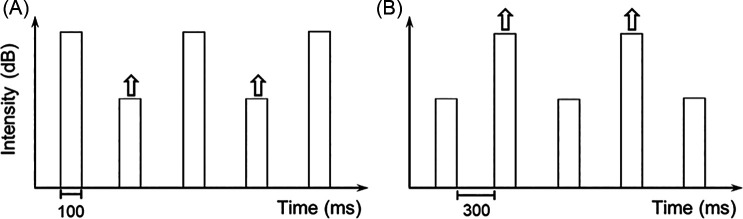
Schematic of two stimuli tested. (A) Low target (LT) stimulus, where an increment in level (indicated by arrows) is added to the lower-level target sounds in the presence of three higher-level nontarget sounds. (B) High target (HT) stimulus, where an increment is added to the higher-level target sounds in the presence of three lower-level nontarget sounds.

A single-interval, yes-no, intensity discrimination procedure was used. Figure 1 is a schematic of the stimuli used, plotting stimulus intensity as a function of time. Each stimulus was composed of five 100-ms sounds with 5-ms cosine-squared onset and offset ramps, separated by 300 ms. In experiment 1, the standard stimulus (i.e., no signal present) had sound levels that alternated between 70- and 35-dB sound pressure level (SPL), i.e., 70-35-70-35-70-dB SPL [Fig. 1(A)]. This series of levels will be referred to as the low target (LT) stimulus because the 35-dB SPL sounds were the target. In experiment 2, *LT* [Fig. 1(A)] and high target [HT; Fig. 1(B)] stimuli were tested. For the HT conditions, sounds had alternating levels of 35-70-35-70-35-dB SPL, and the intensity increment was applied to the 70-dB SPL sounds.

The magnitude of the level increment (as Δ*L* in dB) was adjusted using a two-down, one-up adaptive procedure (Levitt, 1971). The initial value of Δ*L* was 3.5 dB, and the step size was 0.5 dB; after four reversals, the step size was reduced to 0.25 dB and the track continued until an additional eight reversals were completed. The 71% correct threshold is the arithmetic mean of the last eight reversals. The minimum value of Δ*L* was set to 0 dB to prevent negative values. In the *baseline* conditions, only the target sounds were present; the first, third, and fifth sounds were replaced with temporal gaps.

### 2.3 Experiment 1

For experiment 1, the stimuli were drawn from Oh *et al.* (2022). The target sounds were the 1st–16th harmonics of 250-Hz fundamental frequency (*f_o_*) and the slope of the spectrum that fell at a rate of −1 dB/octave with phases drawn randomly prior to each stimulus presentation. The characteristics of the nontarget sounds varied to form different conditions. In the *same* condition, the target and nontarget sounds had the same fundamental frequency and harmonic numbers. In the *low*, *lower*, and *lowest* conditions, the *f_o_*'s of the nontarget sounds were 198, 176, or 136, respectively, with no harmonics presented above 4000 Hz, and the slope of the spectrum fell at −1 dB/octave. In the *steep*, *steeper*, and *steepest* conditions, nontargets were the 1st–16th harmonics of *f_o_* = 250 Hz, and the slope of the spectrum fell at a rate of −1.9, −2.5, or −7 dB/octave, respectively. The values for the fundamental frequencies and spectral slopes were chosen from Oh *et al.* (2022), where the smallest changes did not uniformly lead to stream segregation while the two larger changes did.

The protocol for experiment 1 was as follows. Initially, listeners practiced in the *baseline* condition. The first five thresholds were averaged. If the average was greater than Δ*L* = 2 dB, another five thresholds were estimated. If the listener's threshold remained above Δ*L* = 2 dB after 2 h of practice, the listener was excluded from the study. For experiment 1, two subjects were excluded. Next, listeners ran the *baseline* condition. After completion, the listeners ran the remaining conditions in random orders. For the initial non-*baseline* condition encountered by a listener, 13 threshold estimates were collected; otherwise, 10 threshold estimates were collected. The last eight threshold estimates are split into two blocks of four to test for practice effects (one-tailed *t*-test). If no practice effects were noted, the average of those eight threshold estimates formed the threshold estimate. On four occasions, practice effects were observed. In these cases, an additional five threshold estimates were collected and the last eight were averaged to estimate threshold.

### 2.4 Experiment 2

Experiment 2 is divided into two parts: experiment 2A and experiment 2B. For experiment 2A, the targets were the 1st–15th harmonics of a 250-Hz fundamental frequency. Three conditions were tested in addition to the *baseline* and *same* conditions. In the *noise* condition, the nontarget sounds were broadband Gaussian noise (after Lutfi and Jesteadt, 2006). The noise was not low-pass filtered prior to headphones. In the *low* and *lower* conditions, the nontarget sounds were the 1st–15th harmonics of *f_o_* = 198 and 176 Hz, respectively. Note that because the harmonic numbers are the same, higher fundamental frequencies yield stimuli with wider bandwidths. In experiment 2B, the targets were the third–seventh harmonics of a 250-Hz fundamental. In addition to the *baseline* condition, in the *off-freq* condition, the nontarget sounds were the 10th–14th harmonics of a 250-Hz fundamental, i.e., having the same fundamental frequency as the target sounds but higher harmonic numbers.

The protocols for experiments 2A and 2B were as follows. In experiment 2A, three listeners ran the LT conditions and then the HT conditions; for four listeners, the order was the opposite. In experiment 2B, three listeners ran the LT conditions and then the HT conditions; for the other three listeners, the order was the opposite. In other respects, the procedures were parallel to those described for experiment 1. Listeners' practice thresholds were sufficiently low that none were excluded from completing the experiment. On one occasion in experiment 2A and two occasions in experiment 2B, practice effects were observed in the experimental condition and repeated as described above.

The *low* and *lower* conditions of experinents 1 and 2 share the same fundamental frequencies (*f_o_* = 198 and 176 Hz, respectively). In experiment 1, the spectral extent (cutoff frequency) is the same for the target and nontargets sounds in the *low* and *lower* conditions. In contrast, in the *low* and *lower* conditions of experiment 2, the nontarget sounds have narrower bandwidths than the targets (by 780 and 1110 Hz, respectively). Comparing the *same*, *low*, and *lower* conditions across experiments provides an opportunity to evaluate potential cues associated with differences in frequencies *per se* (experiment 1, the spectral extent is similar, but the harmonic components are not) versus changes in spectral regions associated at higher frequencies (experiment 2, same range of harmonic numbers and different bandwidths).

## 3. Results

For experiments1 and 2, the difference in thresholds between the *Baseline* and *same* conditions is expected to be the largest. Relative to that value, threshold differences between the *same* and experimental conditions would reflect a release from level dominance.^2^ Note that the target sounds are unchanged, meaning any shift in thresholds reflects the properties of the nontarget sounds (parallel to Lutfi and Jesteadt, 2006).

### 3.1 Experiment 1

**Fig. 2.**
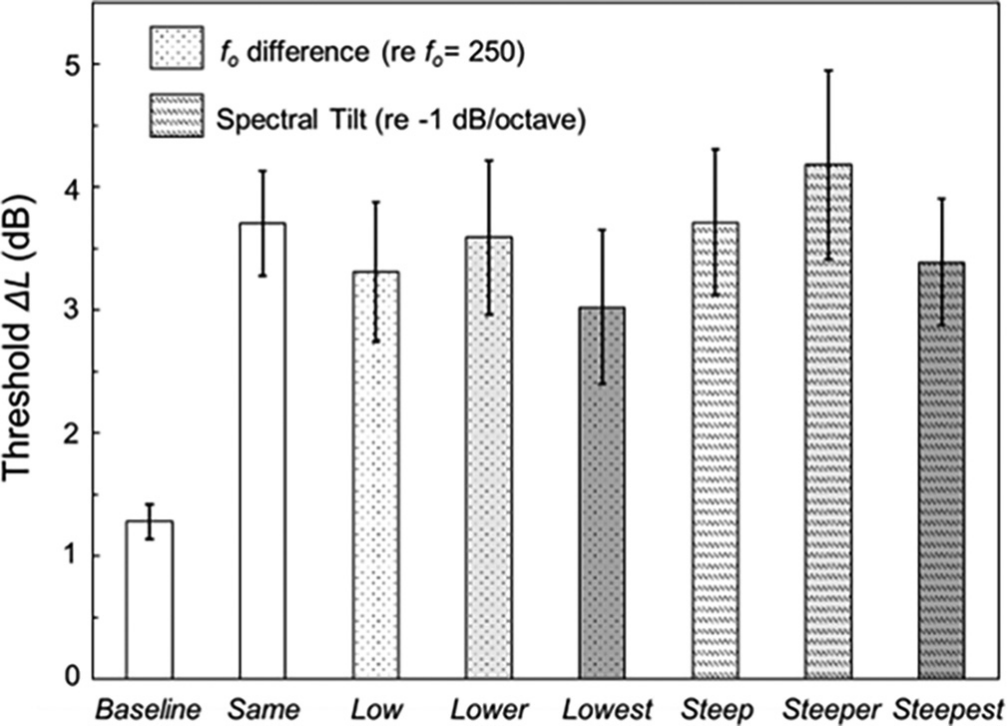
Thresholds for experiment1 as Δ*L* in dB. In different LT conditions, nontarget sounds are absent (*baseline*), nontarget and target sounds are the same (*same*), nontarget sounds have different fundamental frequencies (*low*, *lower*, and *lowest* conditions), or different spectral slopes (*steep*, *steeper*, and *steepest* conditions) relative to target sounds. Error bars indicate ± one standard error of the mean.

Figure 2 plots the mean thresholds (LT) for all conditions; *baseline*, *same*, and the three conditions with differences in fundamental frequency and spectral slope (left to right), smallest to largest change, respectively. As expected, thresholds in the *baseline* condition are lowest. Adding nontarget sounds (*same*) yields a 2.5 dB increase in threshold relative to the *baseline* condition. The thresholds associated with changes in the fundamental frequency and spectral slope of the nontargets do not demonstrate graded releases from level dominance, i.e., there is no consistent reduction in thresholds as the fundamental frequency/spectral slope of the nontarget sounds deviate more and more from the targets. This is counter to the proposed expectations based on primary sound segregation results (Oh *et al.*, 2022).

Potentially, the changes in stimuli tested here do not provide a release from level dominance because the perceptual difference between the target and nontarget sounds is not large enough and, thus, failed to overcome the salience of the higher-level nontargets. To explore this possibility, in experiment 2, target and nontarget sounds differed in terms of the extension of energy in nonoverlapping frequency regions.

### 3.2 Experiment 2

Across conditions in experiment 2A, threshold Δ*L* s for the HT stimulus were within 0.1 dB of each other, including the *baseline* condition. That is, thresholds with and without low-level nontargets were the same regardless of the characteristics of the low-level nontargets (see also Oberfeld, 2008 for parallel results in forward masking).

**Fig. 3.**
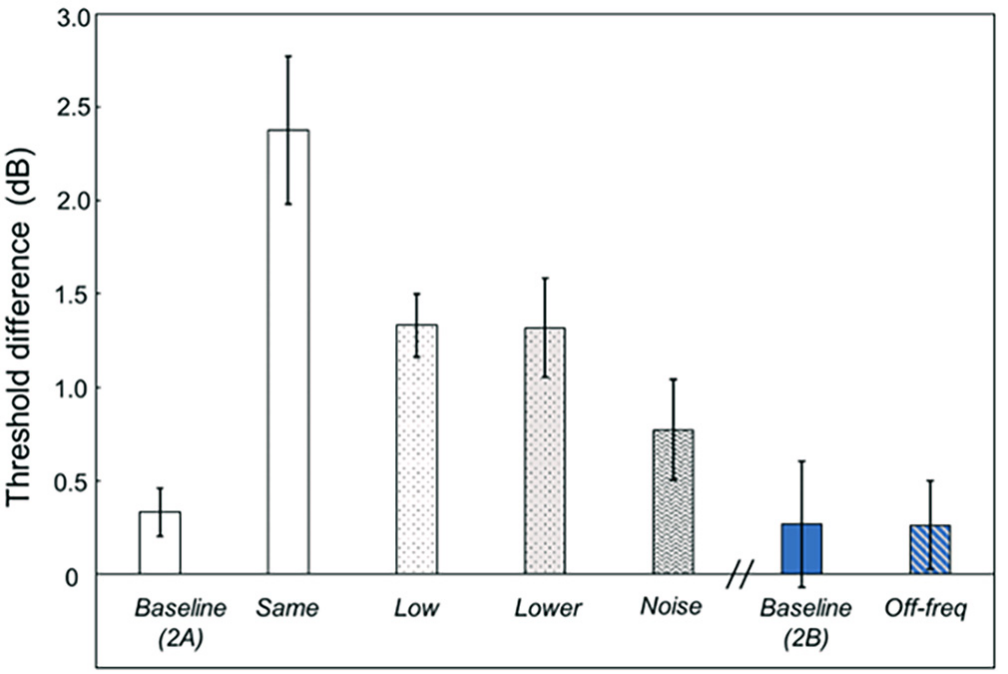
Differences in thresholds, LT minus HT. The first five bars are from experiment 2A, and the rightmost two bars are from experiment 2B. Relative to the target sounds, the nontarget sounds are absent (*baseline*); the same (*same*); of different fundamental frequencies but the same harmonic numbers (*low*, *lower)*; broadband noise (*noise*); of the same harmonic frequency but different harmonic numbers (*off-freq*). Error bars indicate ± one standard error of the mean.

Figure 3 plots the difference in LT thresholds minus HT thresholds. The left side shows results for experiment 2A, and the right two bars are for experiment 2B. The leftmost five bars are *baseline*, *same*, *low*, and *lower* with nontarget fundamental frequencies of *f_o_* = 198 and 176 (1st–15th harmonics), and *noise* (nontargets were noise bursts). The right side shows results for the *baseline* (different target sounds than in experiment 2A) and *off-freq* conditions in experiment 2B. Because thresholds across the HT conditions were essentially identical, Fig. 3 effectively reflects threshold profiles for the LT conditions. Unlike the results of experiment 1, in the current experiment with equal harmonic numbers, changing fundamental frequencies improved the thresholds. Presumably, this reflects not a change in the fundamental frequency but a shift in the frequency extent for the target relative to the nontarget sounds. For the *noise* condition, the level dominance was further reduced (as in Lutfi and Jesteadt, 2006). The two rightmost bars in Fig. 3 are for experiment 2B (blue online) in which the target sounds were harmonic complexes with a fundamental frequency of 250 Hz and the third–seventh harmonics. For the *off-freq* condition, the added nontargets shared the 250-Hz fundamental with the targets but were composed of the 10th–14th harmonics. When the nontarget sounds and target sounds had distinct regions of activation, a threshold difference was essentially identical to *baseline*, i.e., no level dominance was observed.

Experiment 2 demonstrates that when the target and nontarget sounds do not occupy the same frequency regions, level dominance is reduced. For experiment 2A, the noise nontarget, which was broadband, provided a release from level dominance. For experiment 2B, in the *off-freq* condition, where the target and nontarget sounds occupied wholly different frequency regions, thresholds for low- and high-level targets were approximately the same. Comparing the results for *low* and *lower* conditions across the two experiments, a release from level dominance occurred when the target and nontarget spectral extents differed, as in experiment 2A, but not when they essentially shared the spectral extent, as in experiment 1. This indicates that relatively small local differences in harmonic frequencies between targets and nontargets do not lead to changes in thresholds, which is consistent with past research (e.g., Zeng and Turner, 1992). Regarding the release of level dominance, we failed to uncover systematic effects of changes in quality. The current results are consistent with mechanisms that are largely peripheral: changes in spectral range but not differences in the spectral shape and fundamental frequency led to changes in thresholds.

## 4. Summary

In the current study, the effects of sound segregation cues on multi-sound intensity discrimination were explored. The data do not support that changes in stimuli which encourage sound segregation, in general, provide a release from level dominance. Changes in fundamental frequency and spectral shape did not release level dominance when the target and nontarget sounds shared the same frequency regions. Regarding changes in the degree of frequency non-overlap between the target and nontarget sounds, the data indicate a somewhat graded release from level dominance with reductions in the amount of spectral overlap. Moreover, makeup of the lower-level sounds did not impact intensity discrimination thresholds for the higher-level sounds, indicating the strong salience of the higher-level sounds.

## Data Availability

The data that support the findings of this study are available from the corresponding author upon reasonable request.

## Footnotes

^1^
Although the K.W. was aware of the manipulations, his data were not noticeably different from the other observers in trend or magnitude. This likely reflects that K.W. did not practice substantially more than the than the other listeners.

^2^
For ease of description, the phrase “release from level dominance” in this paper refers to reduction in thresholds in experimental conditions relative to the *same* condition.
